# Statewide Ambulance Coverage of a Mixed Region of Urban, Rural and Frontier under Travel Time Catchment Areas

**DOI:** 10.3390/ijerph18052638

**Published:** 2021-03-05

**Authors:** EunSu Lee, Melanie McDonald, Erin O’Neill, William Montgomery

**Affiliations:** 1 Management Department, New Jersey City University, Jersey City, NJ 07311, USA; mmcdonald@njcu.edu; 2 Health Sciences Department, New Jersey City University, Jersey City, NJ 07305, USA; eoneill@njcu.edu; 3 Earth and Environmental Sciences Department, New Jersey City University, Jersey City, NJ 07305, USA; wmontgomery@njcu.edu

**Keywords:** service coverage, GIS, population covered ratio, land coverage, backup service, rural public health, response time, chute time, catchment

## Abstract

This study examines the statewide service coverage of emergency medical services (EMS) in view of public health planners, policy makers, and ambulance service managers. The study investigates the statewide service coverage in a mixed region of urban, rural, and frontier regions to address the importance of ambulance service coverage at a large scale. The study incorporated statewide road networks for ambulance travel time, census blocks for population, and backup service coverage using geographic information systems (GIS). The catchment areas were delineated by the travel time after subtracting chute time for each Census Block as an analysis zone. Using the catchment areas from the ambulance base to the centroid of Census Block, the population and land coverage were calculated. The service shortage and multiple coverage areas were identified by the catchment areas. The study found that both reducing chute time and increasing the speed of emergency vehicles at the same time was significantly more effective than improving only one of two factors. The study shows that the service is improved significantly in frontier and urban areas by increasing driving time and chute time. However, in rural areas, the improvement is marginal owing to wider distribution than urban areas and shorter threshold response time than frontier areas. The public health planners and EMS managers benefit from the study to identify underserved areas and redistribute limited public resources.

## 1. Introduction

Since the 1960s, Emergency Medical Service (EMS) care has increased its geographic reach to cover the entire United States [1]. Response time is widely being evaluated to measure EMS vehicles’ performance in a variety of perspectives, even though it is a controversial performance measure being used for all categories of incidents. Delayed response by emergency vehicles will negatively affect patient outcomes to the higher priority EMS calls, which are “time sensitive” and potentially life-threatening incidents [2]. Local government or community may contract with ambulance suppliers to provide services to the community, thereby setting target average response times [3]. Response time can be a useful performance indicator for EMS ambulance service design and easily perceived by the public [4]. Therefore, it is of critical importance that the dissemination of available ambulances allows for timely responses. EMS agencies serve patients in various regions ranging from urban settings to locations in rural areas. While much more is known about ambulance coverage for urban areas, there are insufficient and unequal pre-hospital services in rural and remote regions [5,6,7,8]. Emergency calls and the need for emergency medical providers are growing, especially in rural and remote communities. Rural and frontier areas are especially challenging to respond to the calls because of extended distances, sparse populations, increased costs, and shortage of healthcare resources [7,9,10,11,12]. Efficient and effective resource management is needed to maximize potential coverage without compromising response time, while service quality and timeliness are improved.

There has been much research on ambulance capacity and scheduling, especially in urban areas, but there is a gap in exploration on statewide planning. With the growth in population across the state of North Dakota (ND, USA) and with increased economic and social activities in Western North Dakota due to the boom of oil exploration and associated activities, a redesign of service coverage may be required. Due to its diverse geographical characteristics of the state of North Dakota, this study investigates a statewide service coverage model with a case study. The state’s land is categorized into urban, rural, and frontier, with a substantial portion of the state recognized as rural and frontier areas. Our model investigates the statewide service coverage based on regional types of urban, rural, and frontier to address the importance of the ambulance location services of the state using geographic information systems (GIS).

This study has three aims: to discover the current coverage with the existing ambulance facilities with travel time catchments considering driving time and chute time; to determine the county level of coverage ratio in terms of population and land (i.e., 90% or 95% coverage); and to visualize back-up service coverage areas to identify underserved areas. Thus, this study can demonstrate a statewide service coverage analysis to meet the legislator’s recommendations of service response time.

The development of a model using the proposed methods is to discover better service coverage and visualize response time aims to improve service quality, timeliness, and efficiency. The discussed approach provides a model to improve emergency management systems in response to population growth and changes in transportation networks and landscapes. The method used in this study can be applied to other states adopting similar policies.

## 2. Literature Review

**Figure 1:**
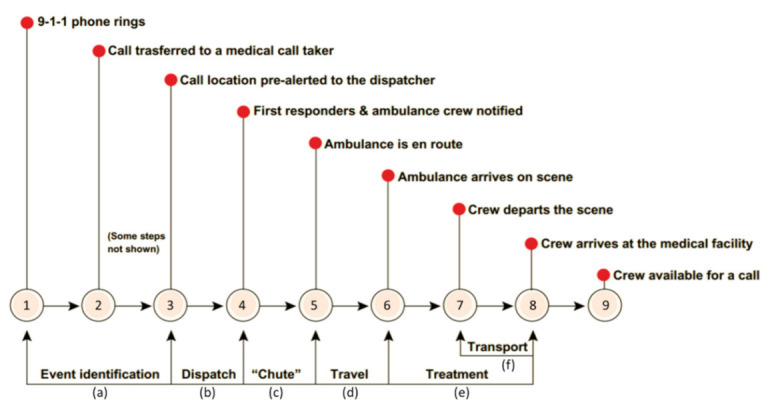
Events and stages of EMS mission time [13].

This literature review discusses three sections: a mixed geographic region, population and land coverage, and geographic backup coverage. Each section is summarized with the contribution of the study to the current literature. For effective communication throughout the paper, the nine events of a typical EMS response time and six stages of the events are explained (Figure 1). 

There are nine events to a typical EMS response: (1) an emergency call is prepared, (2) the call is transferred to a medical call taker, (3) the medical call taker notifies call location to the dispatcher, (4) first responder(s) and ambulance crew is notified, (5) an ambulance is en route, (6) the ambulance arrives on scene, (7) a crew departs the scene, (8) the crew arrives at the medical facility, and (9) the crew is available for a call. The events flow through the following six stages: (a) event identification from event (1) to event (3), (b) dispatch between (3) and (4), (c) chute between (4) and (5), (d) travel between (5) and (6), (e) treatment between (6) and (8), and (f) transport between (7) and (8) [13]. The stage of travel is actual driving time. In this study, response time refers to the stages (b) and (c).

### 2.1. A Mixed Geographic Region 

To determine the service coverage of an optimization problem, models use distance as a generalized cost from the ambulance facility to the demand area [14], which is usually the centroids of the populated district (e.g., census block) [15]. In addition to the distance, the models also take into account the traffic characteristics of the roads, such as traffic volume and travel speed for response time [15]. This analysis of the response time considering the chute time practically is more descriptive [16,17]. A geographic region of urban and rural affect the ambulance service. In urban areas, traffic on roads deteriorates the travel time [12], and frequent calls require multiple units and staff. On the contrary, in rural and remote settings, longer distances and lack of capacity diminish service quality. Therefore, classifying geographic regions in the EMS service coverage analysis is a necessary preprocess to understand the EMS service in a mixed region. 

In general, the ambulance service coverage problem adopts an equal travel time catchment or minimizes travel time. Baket et al. [6] redesigned the primary response coverage for county EMS by balancing travel time to provide equitable delivery of services to the community. He et al. [5] evaluated a statewide rural EMS by measuring the service coverage ratio index and the service timeliness index. The study applied a 15 min threshold of driving time for the entire state of South Dakota. Cho et al. [12] conducted a case study of Seoul, Korea to characterize the influence of transportation infrastructure of urban EMS. The study delineated k-minute coverages using driving time from EMS stations to the centroids of populated grids (100 by 100 m). Tansley et al. [18] conducted a case study of the national ambulance service of Ghana. The study measured the population covered by catchment areas of 30 and 60 min of driving time distance. 

The EMS demand and service capacity are different in urban and rural regions [7,8]. Berg et al. [7] conducted a case study of the Vestfold region in Norway by classifying demand points into urban and rural. The coverage threshold of 12 and 15 min response time were used for urban and rural regions, respectively. The study used a maximum travel time by subtracting median prep-trip delay (i.e., Chute time) from the coverage threshold response time. Lee [19] analyzed service coverage for a mixed region of urban, rural, and frontier with a service coverage threshold of 9, 15, and 20 min in parts of North Dakota. The study utilized the ZIP code areas and estimated service levels of ambulances based on driving time as response time without considering chute time. Ulteig [17] analyzed the service coverages in the oil and gas counties in North Dakota. The study adopted the response time threshold of 20 min by considering the counties as rural. The mobilization time (i.e., chute time) of 5.3 min was subtracted from 20 min to estimate actual driving time from ambulance stations to the scenes. 

From the literature, it is found that mixed geographic regions are applied on a local or regional scale. Recent studies have been applying actual driving time to analyze EMS service coverage with single or double geographic regions. However, it is still not well-reviewed on a larger scale for the state level with mixed geographic regions. 

### 2.2. Population and Land Coverage

Population density data is not recommended to predict the number of incidents for air ambulance service [20]. Since population density and the 911 calls are not strongly related, studies also utilized historical incidents. However, the historical data is not available from all zones, and long-range planning should address equitable service to a community [6]. Equitable service requires all residents experience equal access to service. Probability-of-coverage maps can help planners diagnose and improve EMS performance [16]. Thus, service coverage analysis for ground ambulance utilizes the population data as a proxy of EMS demand. The EMS plan, which responds to locally distributed demographic-based demands, recognizes the ambulance facility as the base location of the responders. Tansley et al. [18] measured population-level spatial access with the station-level ambulance to population ratio.

Hogan and Revel [21] estimates the service level by calculating the population served by the primary, secondary, and tertiary coverage area. Likewise, Lee [19] also used a population to estimate an index of demand covered ratio in a view of ambulance users. Cho et al. [12] measured both area coverage and population coverage within in k-minutes. The study found a significant variation in 5 min coverage for both area and population coverage by district (i.e., zone). The variation is obvious and not always proportional so analyzing for each zone is crucial for ambulance service planning. 

We found that the population is widely used as a proxy of demand and measures the population covered by the service. Since many zones do not carry historic calls [7], the zones should estimate the 911 calls for further analysis. For that reason, the area coverage can be utilized [12]. However, the literature shows that the areas covered have been ignored. Thus, this study investigates the population and land coverage to understand the distribution pattern of ambulance facilities and service coverage of the service providers.

### 2.3. Geographical Backup Coverage

When it comes to the ambulance backup service model, it is categorized into the ambulance backup service problem with multiple units and geographical backup coverage problem. A facility, which has multi-units with a significant budget and high demand, can simultaneously respond to multiple calls. 

With the ambulance backup service problem, the multiple service is designed using a queueing theory to manage EMS resources efficiently. This facility operates one or more ambulances and emergency medical personnel based on whether the ambulance staff are full time or part time staff or volunteers, providing limited services. In general, one or more emergency vehicles serve an area. If for some reason the facility cannot respond to the calls exceeding its operational capacity, the call(s) can be covered by the next closest facility, which is called geographical backup coverage. This is ambulance station-based backup coverage. It is common practice to search for optimal solutions to the availability of ambulance facilities and staff, depending on the budget or service level given [22]. Most of the problems mentioned above include optimization theory to determine the optimal or the next best ambulance location or service level. In the EMS plan associated with ambulance managers, policymakers, and service planners, taxpayers need to understand the current situation and service levels accurately [23]. 

For example, Hogan and Revelle [21] minimized the number of ambulance facilities for each call to be covered by the third backup service. Each call should be covered by at least three nearest ambulances while minimizing the total number of ambulances. It is assumed that the vehicle is dispatched from the nearest ambulance facility to respond to a call. Therefore, this study recognizes the presence of one or more ambulances in an ambulance facility as a single location and uses them for planning coverage of service. If service is unavailable due to a simultaneous response to the previous demand(s) in the same area or due to a lack of staff in operations, one of the other nearest facilities is assumed to be dispatched. In this case, a maximum service coverage problem solves the geographical backup coverage [14].

How many ambulances may cover an area? This is not answered yet. Thus, this study can analyze and visualize the number of catchment areas overlaid over a region and identify underserved areas.

### 2.4. Summary 

This section reviewed the spatial distribution, population-level spatial access, and geographical backup coverage. To the best of the authors’ knowledge, no research has been conducted to present multiple-catchment floating areas by statewide analysis simultaneously considering actual interactive drive time by offsetting the chute time. Therefore, this study will address this gap in research and provide insightful information for county planners of a state. With this information, state planners can design service coverages and redistribute limited EMS infrastructure resources across the state to balance the ambulance services.

## 3. Model Development

Two performance indicators are measured to improve the system: (1) population- and land covered ratio and (2) response time for each analysis zone.

### 3.1. Regional Service Model Using Geographically Different Response Time

Due to limited resources, all demands cannot be covered to meet the requirement. However, theoretically 95% of the population should be included in the location and resource allocation since the United States Emergency Medical Services Act of 1973 set a value of 10 min for the basic service with a 95% service level (α). Therefore, the proportion of population constraint (1) can be added to the previous set-coverage and facility location problems.

(1) $$\underset{i}{\sum }h_{i}z_{i}\geq \alpha \underset{i}{\sum }h_{i}\;$$

where *h_i_* = the population of an analysis zone (e.g., census block, township, or county) *i*

$$z_{i}=\left\{\begin{array}{ll} 1 & \mathrm{if}\;\mathrm{demand}\;\mathrm{node}\;(\mathrm{centroid}\;\mathrm{of}\;\mathrm{area})\;i\;\mathrm{is}\;\mathrm{covered}\\ 0 & \mathrm{otherwise} \end{array}\right.$$

However, if the service coverage is being considered for diverse types of areas such as urban/city (a.k.a. urban), rural/suburban (a.k.a. rural), and frontier/remote (a.k.a. frontier), the service level should apply to each region (2).

(2) $$\underset{i\in R}{\sum }h_{i}z_{i}\geq \alpha \underset{i\in R}{\sum }h_{i}\;,\;for\;R=\left\{0,1,2\right\}$$

where,

$$R=\left\{\begin{array}{l} 0,\;\mathrm{Urban}\;\\ 1,\;\mathrm{Rural}\;\\ 2,\;\mathrm{Frontier} \end{array}\right.$$

For example, the North Dakota Department of Health (NDDoH) testified to the Public Safety Committee to uphold a service response rate (α) of 90% under the service response times as follows [24]:

- 9 min in urban areas (*R* = 0);
- 20 min in rural areas (*R* = 1);
- 30 min in frontier areas (*R* = 2).

### 3.2. Response Time: Chute Time and Travel Time

In the previous section, it was assumed that the ambulances which received emergency calls are dispatched from their garages (i.e., bases). However, ambulances roam somewhere in the service area so that the ambulances can respond to any emergency calls quickly in metropolitan areas (e.g., Fargo in North Dakota). In that case, the dynamic locations can be applied for stochastic coverage analysis as catchment area analysis [19].

The major urban areas in North Dakota are served by ambulances which carry full time paramedics and *en route* ambulances, while rural and frontier regions heavily rely on volunteer first responders. Therefore, before drawing the service coverage, we should understand the mobilization time of crews (i.e., chute time) has an impact on the service level of response time required by state legislators. In other words, the required response time of 9 min in urban, 20 min in rural, and 30 min in frontier areas will be deteriorated by the chute time. To estimate the chute time, the average chute time was downloaded for the Midwest region from the National Emergency Medical Services Information System (NEMSIS) Database [25]. The data are not available at the state level, but regional data of the Midwest are accessible. A two-year period, 2014–2015, was analyzed to understand the chute time in Midwest for ND. Each sample is an aggregation of a daily activity report. Thus, each sample size varies day by day. In addition, the chute time is not gathered from dispatch centers based on time stamps of radio calls but on “self-reported” times gathered from electronic patient care records (PCR). Only urban systems using a computer aided dispatch (CAD) that report times directly to the PCR will be completely accurate. 

The data was extracted for the Midwest because the state of North Dakota is located in this region. NEMSIS categorizes the areas into urban/city, urban/suburban, rural, and wilderness (i.e., frontier), and the organizations status as volunteer and non-volunteer. The difference in volunteer vs. non-volunteer EMS corps is compensation. EMS professionals who volunteer do not receive pay (or, in rare cases, nominal pay), but they can receive continuing education opportunities [26]. The level of services includes EMT-intermediate, EMT-paramedic, first responder, and other agency values (excluding physician and nurse).

The median and the 95% confidence level of chute time in the Midwest region are summarized in Table 1 based on the NEMSIS database. The median response time for the ambulance with volunteers in urban/suburban areas was 4.87 min, while the median value for the non-volunteer (i.e., full-time staff) was 2.50 min. On the other hand, the median chute time for a volunteer ambulance in a wilderness area (the frontiers of North Dakota), is estimated at 6.25 min, and at a 95% confidence level, between 6.13 min and 6.33 min.

### 3.3. Performance Measure: Population- and Land Covered Ratio

The study of ambulance service coverage requires appropriate measurement for mapping out the underserved areas (i.e., service shortage area) and estimating the population covered under the level of service (i.e., population-covered-ratio). This study measures current performance based on the present ambulance bases with the average travel time from any closest ambulance location(s) to the centroid of the populated areas (i.e., census blocks) covered by the recommended service response time (catchment area) and the total populations covered by the current ambulance service locations.

Total population covered by the current locations and policy is one of the major concerns for policy makers and taxpayers living in the study areas. The covered population is dictated by the threshold response time ($RT_{0}$) required by the state legislators. The threshold response time varies by regions ($RT_{0}^{R}$) of urban, rural, and frontier, expressed as “*R*” in this study. Performance is measured by population and land covered for the statewide population for a statewide model (3) and each region in a regional model (4). Equation (4) can calculate what percentage of the total population in each county (*P*_c_) is serviced by the nearby ambulances if the Census Block in the county (c) can be serviced within the required response time (5).

Response time of an ambulance (*j*) to the centroid of a census block (*i*) is expressed as *RT_ji_* (6). Therefore, the response time should be a sum of travel time from ambulance facility to the scene (*d_ji_*) and chute time (*CT_j_*). In other words, the ideal travel time to the scene (*d_ji_*) subtracts chute time (*S_j_*) from the recommended response time *RT_ji_* (7). If the census block (*i*) is covered by at least one ambulance service based on the regional threshold response time ($RT_{0}^{R}$), the response time (*RT*) to the centroid of a census block from at least one of the ambulance locations (*j*) is the value of one; otherwise, it will be zero (see Equation (8)).

(3) $$P\left(\%\right)=\frac{\sum_{i}h_{i}z_{i}\;}{\sum_{i}h_{i}\;}\times 100$$

(4) $$P_{R}\left(\%\right)=\frac{\sum_{i\in R}h_{i}z_{i}}{\sum_{i\in R}h_{i}\;}\times 100$$

(5) $$P_{c}\left(\%\right)=\frac{\sum_{i\in c}h_{i}z_{i}\;}{\sum_{i\in c}h_{i}\;}\times 100$$

(6) $$RT_{ji}=d_{ij}+CT_{j}\;$$

(7) $$d_{ji}=RT_{ji}-CT$$

(8) $$z_{i}=\left\{\begin{array}{c} 1,\;if\;RT_{ji\in R}\leq RT_{0}^{R}\;\\ 0,\;if\;RT_{ji\in R}>RT_{0}^{R}\; \end{array}\right.$$

where, 

*i* = a census block, *i* = {1, 2, …, *I*};

*j* = an ambulance facility, *j* = {1, 2, …, *J*};

*R* = a geographic region, *R* = {0, 1, 2};

*c* = a county, *c* = {0, 1, … C}.

*P_R_* (%) = the ratio of population covered by the existing ambulance locations *j* over the total population of each region *R*;

*P_c_* (%) = the ratio of population covered by the existing ambulance locations *j* over the total population of each county *c*;

*P* (%) = the ratio of population covered by the existing ambulance locations *j* within the required service time over the total population of the state regardless of regions;

*d_ji_* = fastest travel time from an ambulance location *j* to centroid of a census block *i*;

*CT_j_* = Median chute time (i.e., set-up time) of an ambulance *j*;

*RT*_0_*^R^* = Threshold response time required for a census block located in each region *R*.

## 4. Case Study

The proposed model was applied to the state of North Dakota. For modeling, there are three key elements: (a) defining urban, rural, and frontiers, (b) assigning travel speed of an ambulance over the roads, and (c) determining chute time depending on region and organization status. The key element (a) is case-specific in Section 4.2, while (b) and (c) are scenario specific in Section 4.5.

### 4.1. North Dakota

This study conducted a case study of the state of North Dakota among the borders of Minnesota, Montana, and South Dakota in the Upper Great Plains. The state also borders the Canadian provinces of Manitoba and Saskatchewan. The population in North Dakota was 642,200 in 2000 and 672,591 in 2010 indicating approximately 4.7% growth rate in 10 years. The state is 69,000.8 square miles in 2010 in area, which is the 18th largest state in the United States. There is a discrepancy of the land size between the U.S. Census Bureau’s quick facts and the sum of areas of Census Blocks of 2010. This study assumes that the size of the state is 70,713.6 square miles. The population density of the state is 9.7 per square mile, indicating that the state is one of the less populated states in the country. Metropolitan cities (including Fargo and Grand Forks in the east, and Bismarck, the state capital) are categorized as urban areas. The major industry of Western North Dakota, which is of lower population density, is livestock, farming, mining, and oil and gas, while agriculture serves as the main industry for the eastern and mid regions of the state. 

### 4.2. Defining Urban, Rural, and Frontier Areas

**Figure 2:**
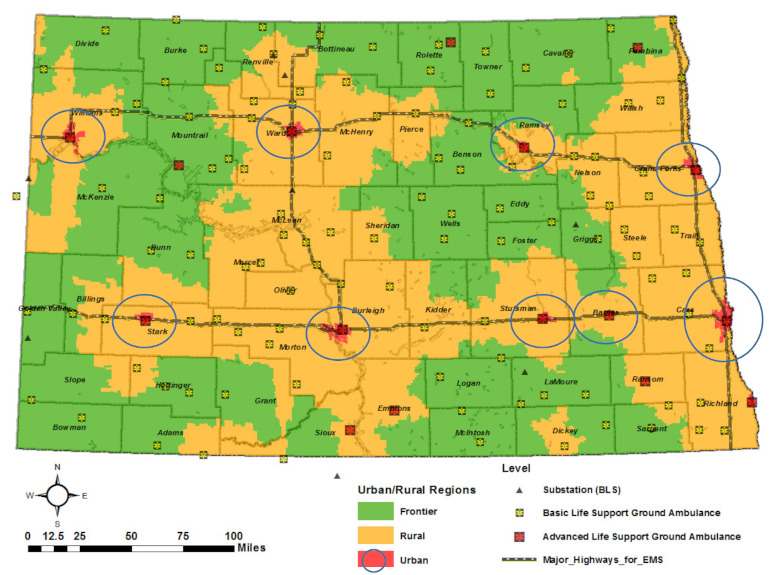
Urban, rural, and frontier regions by Census Block in North Dakota.

“Urban” areas are defined as areas that are densely populated by large groups of people in a manner that is built up. Conversely, areas that are not urban are defined as “Rural” as per the 2010 Census and American Community Survey [27]. For that reason, defining the frontiers in North Dakota is necessary for the investigation of reasonable service coverage. While urban and rural areas are defined at the Census Block level, frontier can be defined based on the purpose of projects being funded [28]. Using ZIP Code Tabulation Areas, the National Rural Health Association (NRHA) defines frontiers with population densities equal to or less than 11 persons per square mile as used in California. However, the frontier and remote area (FAR) codes describe territory characterized by some combination of low population size and high geographic remoteness [29]. The FAR codes were downloaded in the format of Excel, and joined to U.S. ZIP Code Tabulation Areas, and then mapped in ArcGIS. The ZIP Code Tabulations Areas are broken into Census Blocks (Figure 2).

This study defines the urban, rural, and frontier areas for the statewide ambulance coverage analysis. An urban area includes only city boundaries of Fargo, Bismarck, Grand Forks, Minot, Valley City, Jamestown, Williston, Dickson, and Devils Lake. Rural areas define the area combining urban and rural using FAR codes and excluding the blocks of urban areas. Frontier areas are referred to FAR codes. 

### 4.3. Data Sources 

The study requires Census Blocks as ambulance service coverage analysis zones, ambulance service locations (i.e., base), and road networks. The data set of Census Blocks is downloadable from the U.S. Census Bureau’s Website [30]. This study uses 2010 population data. Census Blocks are statistical areas bounded by visible and non-visible boundaries [31]. The Census Block is the finest geographical area for which data are available from the U.S. Census [32]. To estimate the emergency demands (i.e., sources of calls), the U.S. Census Blocks were used. The size of Census Blocks is 0.528627 square miles on average with the standard deviation of 0.259573 square miles. Nonresidential areas consist of 86,210 blocks.

Ambulance service locations are available from the North Dakota GIS Hub Data Portal and was updated in 2017 [33]. The data set does not include alternate posting locations in urban areas. The federal and state highways [34], city and county roads [35], and Topologically Integrated Geographic Encoding and Referencing (TIGER^®^) lines are available from the North Dakota GIS Hub Data Portal. The road data sets are updated frequently. This study used the data sets modified in 2018. 

### 4.4. Scenarios, Assumptions and Parameters

Four scenarios are discussed in this section. Scenario 1 represents the current system without improving the chute time, and driving time is estimated by the speed limit posted and designated by the state on the roads. Scenario 2 assumes that while the vehicle speed increases on the roads, no changes are considered in the chute time. The chute time is improved in Scenario 3, and the vehicle speed remains the same in the scenario. Scenario 4 is to speed up the vehicle on the roads and shorten the chute time.

#### 4.4.1. Travel Speed Estimation

For this study, a standard speed limit was assigned to the TIGER^®^ roads in North Dakota [36]. The speed limits are posted on the roads in North Dakota. However, in the absence of posted speed limits, North Dakota state law designates speeds limits based on road types, access control, and regional situations. Referring to the standard speed limits in North Dakota, this study assigns expected speeds on the TIGER^®^ roads as shown in Table 2. MTFCC (MAF/TIGER Feature Class Code) is a feature class with a five-digit code and describes geographic objects [37]. 

For sensitivity and scenario analyses, various assumptions of the speed limits were applied. For example, Berg et al. [7] took the actual speed if the speed limit on roads is less than 40 km/h, multiplied the driving speed by 1.15 if the speed limit is 40 km/h, and multiplied the speed limit over 40 km/h by 1.2. Tansley et al. [18] increased and decreased base travel speed by 20%. The travel speed assumed for Scenario 2 and 4 add five miles to the estimated speed for Scenario 1 and 3 except for S1500 (Vehicle trail) and S1630 (Ramp). For the ambulance’s safety over S1500 and S1630, the travel speeds remained the same.

#### 4.4.2. Chute Time and Travel Time

Scenario 1 and 2. It was assumed that the chute time of the ambulance facility (*CT_j_*) was 2.5 min in urban areas, 5.5 min in rural areas, and 6.25 min in frontier areas. Therefore, since the recommended response time to emergency calls in the urban areas (*R* = 1) is 9 min (*RT_ji_*), 2.5 min (*CT_j_*) is deducted from the 9 min (*RT_ji_*) to estimate the ideal ambulance drive time of 6.5 min on roads (*d_ji_*). Similarly, if an ambulance located in urban areas respond to calls from rural areas (*R* = 2), the chute time of the ambulance is 2.5 min (*CT_j_*), thus the ambulance should drive to the scene within 17.5 min (*d_ji_*) to meet the recommended response time of 20 min (*RT_ji_*) for North Dakota.

Scenario 3 and 4. The time required for chute time is reduced as shown in Table 3. It assumed that urban areas do not follow the data from NEMSIS, but full-time responders with more resources and better management is assumed to reduce chute time to 0.5 min. Chute times of rural and frontier areas are reduced, but only by 10% from the NEMSIS chute time. For example, the chute time of the rural areas will be assumed to be 4.95 min, down 10% from 5.5 min, while the frontier areas will be reduced 10% in their chute time to 5.62 min. Residents in the frontier areas (*R* = 3) who need emergency services within 30 min (*RT_ji_*) can expect an ambulance located in a rural area to travel within less than 25.05 min (*d_ji_*). Similarly, if residents in a rural area (*R* = 2) make an emergency service request to 911, an ambulance in a frontier area will have to drive 14.38 min (*d_ji_*) because there are no ambulances available in the rural area.

### 4.5. Results and Visual Analytics

#### 4.5.1. Backup Coverages

**Figure 3:**
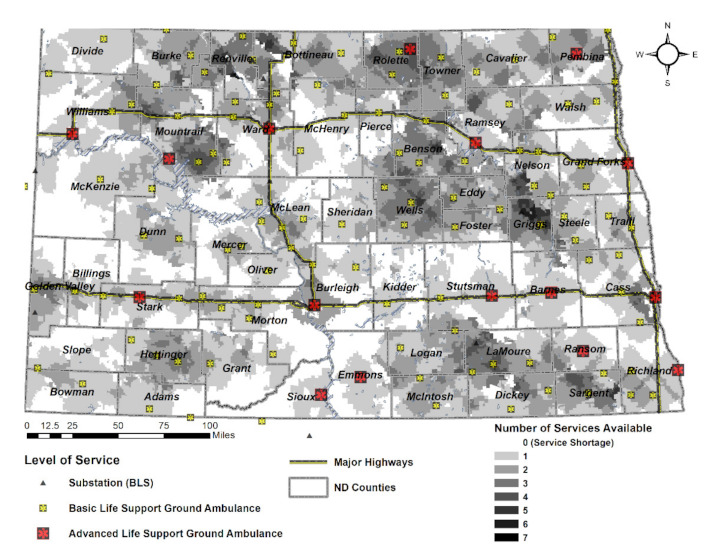
Service map with service shortage (=0) and multiple ambulance services available (≥2) per Scenario 1.

Considering the pre-hospital emergency response time (Figure 3), the analysis of the service coverage according to the emergency response time showed that ambulance coverage with relatively high service concentration is receiving multiple services as expected. What is unique is that it appears to be geographically well-served, mainly in the Northwest, Northeast, and Southeast of North Dakota. In particular, it is analyzed that parts of the Northeast and Midwest can be serviced within the expected response time by more than five ambulance facilities. There are ambulance facilities located close to the highway, but the concentration of ambulances is believed to be low near the highways.

#### 4.5.2. Service Level by Region: Population Covered Ratio and Land Covered Ratio

Table 4 shows how many people are eligible for service in the recommended response time by geographical categories. The results are discussed for each scenario.

Scenario 1 (As-Is): a total of 350,271 people or 91.2% of the population (population covered ratio) in urban areas, can expect an ambulance to arrive within nine min. On the other hand, 87.1% or 159,054 people, in rural areas could receive emergency medical services within 20 min. For the frontier population, 97.6% are expected to receive emergency services within 30 min. It is noteworthy that 87.1% of the population (population covered ratio) in rural areas can be serviced within 20 min, while only 60.0% of the land (land covered ratio) in the areas can expect service within 20 min. It is understood that the population distribution of rural areas is heavily distributed in certain areas. Overall, 91.1% of the population and 74.1% of land of the state of North Dakota will be able to receive services within recommended response time.

Scenario 2 (Increase of Travel Speed): Compared to Scenario 1, the population covered ratio and land covered ratio increased by 1.3% and 5.4%, respectively, based on the EMS response time required in the frontier areas. In rural areas, population covered ratio and land covered ratio increased by 3.3% and 8.2%, respectively. Despite improved land covered ratio, it still falls short of the 90% basic coverage ratio at 68.2%. In urban areas, the coverage ratio rose 4.6% and 10.1%, respectively, in population and land. Overall, it shows a 3.79% increase in population covered ratio and a 6.8% increase in land covered ratio. This is higher than Scenario 3 for all regional service areas and a lower coverage ratio than Scenario 4. The frontier areas seem to have longer response times than rural and urban areas, even if the vehicle speed is increased by 5 mph over the roads.

Scenario 3 (Reduction of Chute Time): In frontier areas, the improvement of population covered ratio and land covered ratio was very marginal by 0.4% and 1.4%, respectively. However, the population covered ratio was 98.0% and the land covered ratio was 89.3%. In urban areas, the improvement of population covered ratio and land covered ratio were 7.2% and 16.8%, respectively. Its improvement is much higher than in rural and frontier regions. The improvement meets the state population covered ratio over 90%, while the land covered ratios still need to be improved. In rural areas, both population covered ratio and land covered ratio still falls short of the state standard of 90% within 20 min even though the improvement of population covered ratio and land covered ratio was 2.2% and 4.7%, respectively. Therefore, in order to provide emergency medical service within the standard response time, the ambulances in rural areas need to increase the speed of emergency vehicle operation and reduce the fleet’s chute time at the same time. Across the state, overall improvement was made by 4.7% and 3.1% for population covered ratio and land covered ratio, respectively. 

Scenario 4 (Increase of Travel Speed and Reduction of Chute Time): Increasing the speed of vehicles and reducing the chute time at the same time can satisfy the standard coverage ratio of population in frontier, rural, and urban areas by 99.0%, 92.7%, and 99.6%, respectively. However, rural areas show a lower coverage than other regions. In light of the land covered ratio, the frontier region shows 94.3%, while the other regions show 72.9% and 88.8%, respectively. 

**Figure 4:**
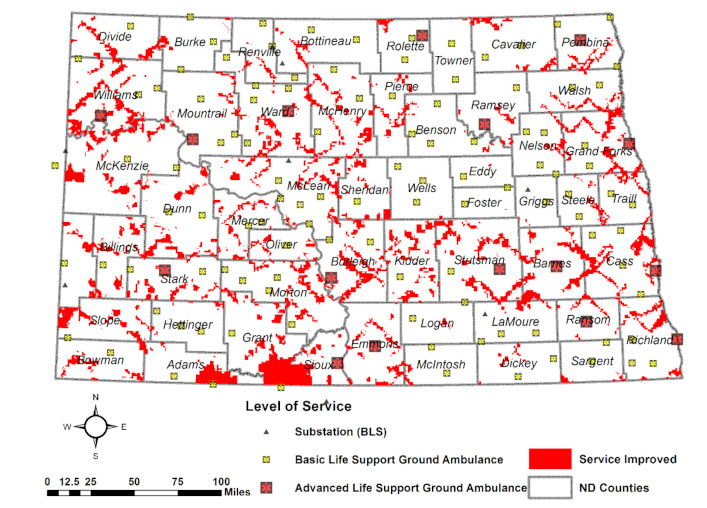
Service coverage improved by increasing travel speed and lowering chute time (Scenario 4 compared to Scenario 1).

Compared with Scenario 1, as a whole, the population and land covered ratios rose 6.5% and 9.7%, respectively, in the frontier region, exceeding the standard-coverage-ratio of 90% over the state. Rural areas show improvements in services, with a 5.6% increase in population and a 12.9% increase in land. More improvement was shown in urban areas than in frontier and rural areas, with 8.4% of the population covered ratio and 11.1% of the land covered ratio being able to receive services within standard service response time, according to the analysis. Census Blocks to receive enhanced services by adopting Scenario 4 are shown in Figure 4. Much improvement was found along the Interstate and U.S. highways.

#### 4.5.3. Service Level by County: Population and Land

**Figure 5:**
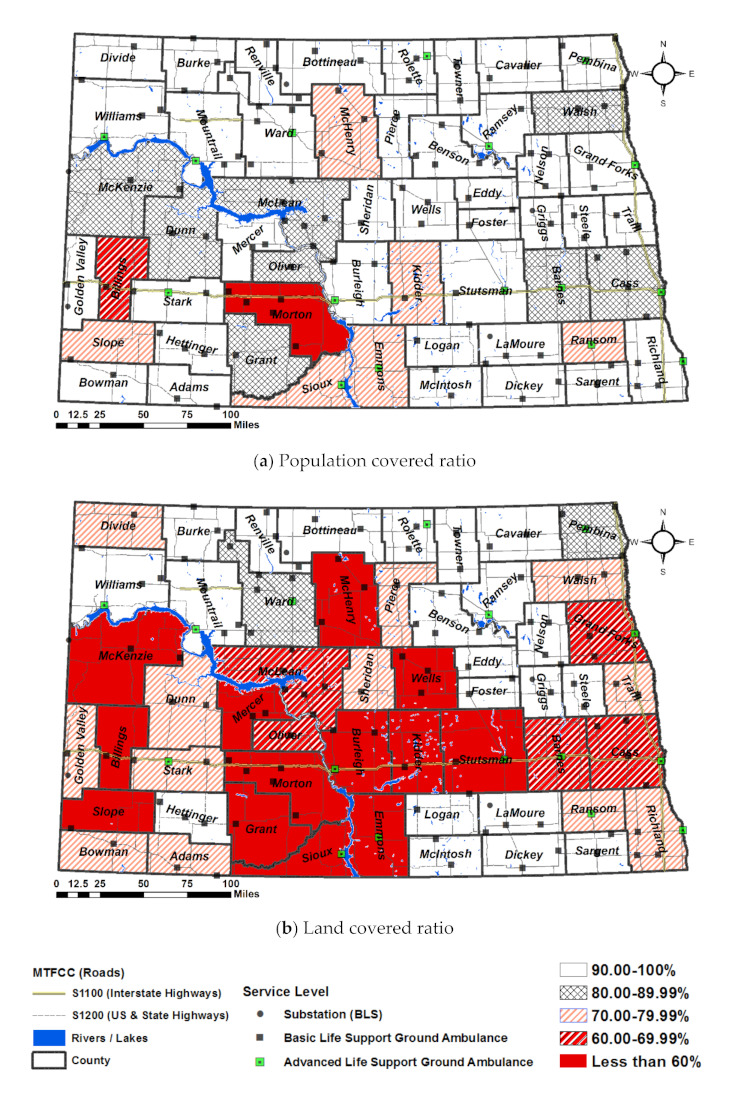
Population covered ratio (**a**) and Land covered ratio (**b**) of Scenario 1.

The results are visualized in Figure 5 with the analysis of Scenario 1 (base scenario of AS-IS). In light of the population covered ratio (Figure 5a), one can expect an ambulance within a standard response time upon a regional service category; for example, only 30.65% of residents in Morton can expect an ambulance in time. Likewise, Billing 62.7%, Kidder 71.29%, Sioux 72.65, Emmons 73.04%, Ransom 74.97%, Slope 75.79%, McHenry 76.81% are less than 80% of the population coverage ratio. Cass, McLean, Barnes, Walsh, McKenzie, Dunn, Oliver, and Grant are falling short of the 90% required by North Dakota.

Most counties that fall short of the service ratio are in the western frontier areas, which include McKenzie, Billing, Dunn, Morton, Grant, Oliver, McLean and Slope or the areas surrounded by the tribal lands include Sioux, Dunn, McLean, Grant, and Ransom (Figure 5b). The counties in the Badlands (McKenzie, Billings, and Dunn) may have lower service ratios due to road deterioration and lack of road connections, which can make it challenging for ambulances to make it to their destination in a timely manner. For the counties in the tribal areas, the response ratios, in general, may be low due to volunteer staff and the lack of funding for staffing, equipment, and training. Response times in tribal areas can also take longer due to greater travel distance required to arrive at the end point as well as insufficient road conditions [11,38,39].

**Figure 6:**
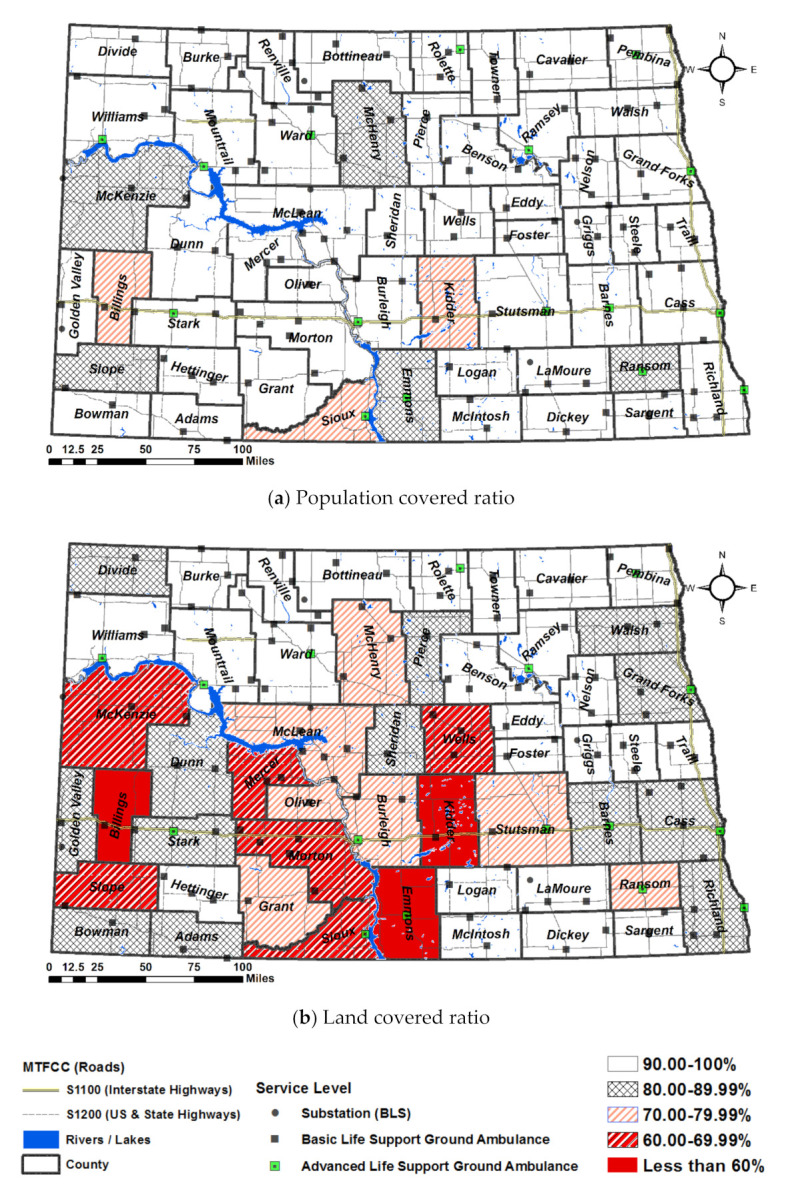
Population covered ratio (**a**) and Land covered ratio (**b**) of Scenario 4.

As Scenario 4 improves the overall service coverage, Figure 6 illustrates the results of the improvement of Scenario 4 compared to Scenario 1 (As-Is). 

By adopting a new management of chute time and travel speed on roads, Morton County experiences up to 62.32% improvement in population served by the local ambulances, thereby covering 92.98% of county population within threshold service response time (Figure 6a). The population covered ratio of Ransom and Emmons Counties has increased by more over 10%, but it is still less than 90%. Sioux, Billings, and Kidder benefit only a small part of improved ambulance service with 76.33%, 71.14%, 79.18%, respectively with the marginal improvement of 3.68%, 8.43%, and 7.89%, respectively. McKenzie, Slope, McHenry, Emmons, and Ransom still does not meet the standard population covered ratio. 

Figure 6b depicts the land covered ratio of Scenario 4. Billings, Emmons, and Kidder are seriously underserved by lower than 60%. In other words, any calls from nonresidential areas might experience slow response. In addition, Sioux, Morton, Mercer, Slope, McKenzie, and Wells are other groups experiencing service shortage. 

Therefore, it suggests finding other ways such as installing an additional station to improve overall service. 

**Figure 7:**
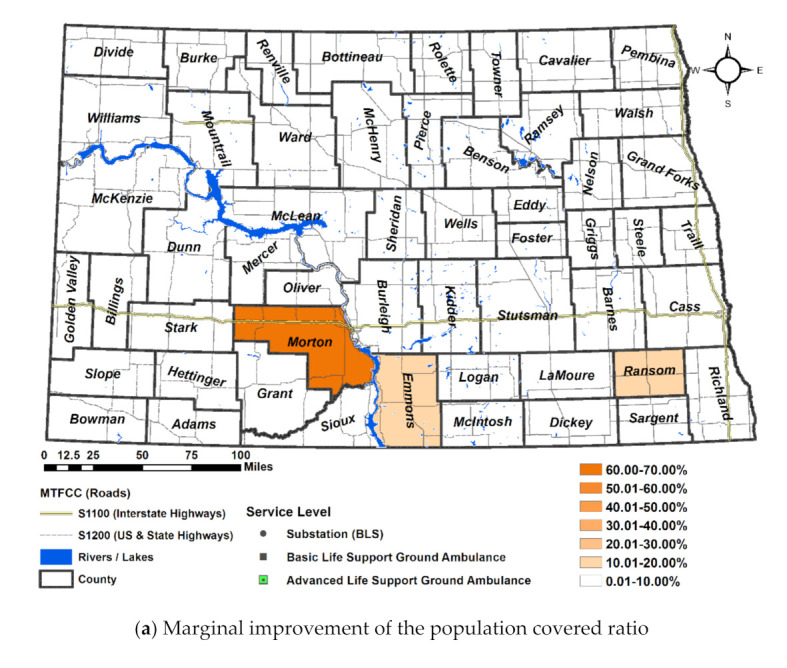

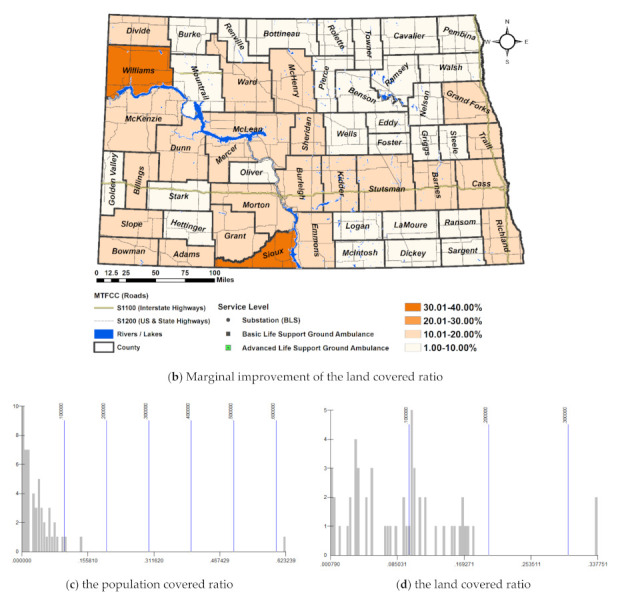
Improvement of Scenario 4 compared to Scenario 1: (**a**) the marginal improvement of population covered ratio, (**b**) the marginal improvement of the land covered ratio, (**c**) the distribution of population covered ratio, (**d**) the distribution of land covered ratio.

By adopting a new management of chute time and travel speed on roads, Morton County experiences up to 62.32% improvement in population served by the local ambulances, thereby covering 92.98% of county population within threshold service response time. The population covered ratio of Ransom and Emmons Counties has increased by more over 10%, but it is still less than 90% (Figure 7a). 

Other significant improvements were shown in Cass 9.51%, Billings 8.43%, Kidder 7.89%, Oliver 6.99%, and Walsh 6.75%. Except Oliver, Interstate 94 and 29 are going through these counties. Cass and Billings show significant improvement than other counties embracing urban areas. Two major cities of Fargo and West Fargo in Cass benefit from the shorter chute time than any other rural counties, which experiences a 10% deduction of the chute time. The other benefit only a small part of improved ambulance service. Therefore, it suggests finding other ways such as installing additional stations to improve overall service (See Figure 7c). 

In Figure 7b, the significant improvement of the land covered ratio is shown in Sioux and William by 33.78% and 33.6%, respectively. The other counties’ improvement are fairly distributed less than 20% (Figure 7d). The other significant improvements of the land covered ratio are found from Adams 18.9%, Kidder 17.06%, Burleigh 16.75%, Grant 17.3%, Grand Forks 15.70%, Cass 15.72%, Billings 13.41%, Morton 12%, McHenry 12.19%, and Divide 10.51%. The other counties show less than 10% improvement.

## 5. Discussion and Implications

### 5.1. Discussion

As predicted, an analysis of all scenarios shows that both decreasing chute time and increasing the speed of emergency vehicles at the same time was significantly more effective than improving only one of two factors. 

The population covered ratio of McKenzie, Billings and Slope Counties in the western region is lower than 90%, and the indigenous and neighboring Grant, Sioux, Emmons, Kidder, McHenry, and Ransom Counties show the population- and land covered ratio of 90% of population, so a plan to provide ambulance services in the region should be devised. Cho et al. [12] find that the reduction in area coverage results in reduction population coverage. The study also emphasized that the magnitude of reduction of area is always proportional to the population coverage. This is a consistent with our results. 

Air ambulance services and other service operation measures should be considered in these remote areas, particularly considering active and growing tourism, economic activities, and social activities. For example, drilling oil wells and mining have increased economic activities [17] and traffic in the non-residential areas over the last decade. In addition, increased accident rates require significantly more emergency services [17].

Even if the current road speed is maintained and the current chute time is required, the state’s recommended 90% of the population covered ratio is met. The urban and remote areas seem to provide services to 90% of the population within the recommended response time of 9 min and 30 min, respectively. However, the regions’ land covered ratio is less than 90%. The ratio does not seem to be important because it is not directly related to the human’s daily lives, but ambulance response time to any accidents from the regions during any activities such as agriculture and recreation can cause great loss due to a delayed response. Even if the land covered ratio is not high, the population covered ratio is high because the population density in urban areas is high and residents in remote areas live close to roads that ensure accessibility. Although rural areas are similar to remote areas, the demand for ambulance response time is shorter, so the population-coverage-ratio seems to be less than 90%.

Scenario 4 assumes a 10% reduction in volunteer ambulance chute time in rural and frontier areas and an ambulance speed of 5 miles faster than the estimated road speed limit. It assumes that the chute time of an ambulance staffed with a full-time employee is 30 s in urban areas, and the travel speed increases 5 mph. This scenario is most similar to the EMS system currently operating in North Dakota. The results of the coverage analysis show that 90% of the population, which is the standard in urban, rural, and remote areas, can receive ambulance services at regional standard time. 

However, in terms of the land covered ratio, the frontier area is covered by 90% of the recommended 30 min, but the remote area is still far from 90%. Downtown areas are also slightly below 90%. As the state’s land covered ratio is less than 90% overall, it is in line with the fact that densely populated areas are limited and land is widely distributed without habitants, just as the state is categorized as a rural state.

Morton County is divided into urban in the city of Mandan and rural areas for the rest of the county. The city of Mandan is served by the ambulance facility in the city of Bismarck, the capital of North Dakota, and the other ambulance facilities are concentrated near the highway, making other areas, along the Missouri River, far from service facilities. Therefore, despite the 20-min response time, ambulance services in rural areas along the river seem to be insufficient within the recommended time. When Morton improved mobilization time and reduced travel times to the scenes, this allowed 90% of the county’s population to be covered within the threshold response time.

On the other hand, counties such as Ransom, Slope, Sioux, Kidder, Billings, McKenzie, and Emmons have lower population coverage than other counties. These counties are the ones which have only one ambulance facility in the county among the counties which are closely bordered by remote areas and are largely classified as rural areas. This single service facility must meet the 20-min threshold of rural response time, and the probability of receiving backup services from nearby counties is also significantly lower. Ambulance facilities, such as McHenry County, are located on the county border, and some places are found to be hollowed out due to lack of backup service coverage in the county’s central area. Therefore, in order to increase the population coverage ratio, one way is to reorganize the area and declare the frontier area, but it is not desirable due to the extended response time. Therefore, it is desirable to provide backup services by sharing ambulance service with neighboring areas. This is the basis of an important argument in the backup service model that previous studies have argued. 

### 5.2. Implications 

Ambulance managers may consider adding in-motion EMS stations in the underserved areas at an operation level. The results of this research have helped fill the gap in literature on this topic and can be used for future research. The visual analytics support the practitioners to implement the public health planning and designing by bridging the academic research. 

This study found that results can assist legislators with a statewide service coverage analysis to aid in the development of policy recommendations for service response time. For instance, the resources of the areas with multiple backup coverage areas can be redistributed to the service shortage areas. With gaps in research on statewide planning, results from this research can be used to develop new policies on speed and chute time especially in rural and frontier areas. 

In addition, public health planners, policy makers, and ambulance service managers should monitor remote areas for changes and development such as economic activities, social activities, and growing tourism, therefore have consensus with the public for the urban, rural, and frontier for this statewide EMS plan.

## 6. Conclusions

Rural and frontier communities are under-served with consideration to emergency medical services (EMS) in unique circumstances. Urban settings also experience deteriorated service due to highly dense populations and urban traffic, and are in need of higher quality service. Thus, understanding the statewide service coverage is crucial to the general public, policy makers, and health designers. Thus, this study investigated the population covered ratio and land covered ratio as part of a gap analysis of public service to address the statewide coverage ratio. 

This study proposed an approach to identify underserved areas with service shortage and the multiple backup coverages for a mixed geographic region of urban, rural, and frontier. The study conducted a statewide service coverage analysis with different drive time from ambulance bases to the demand location of Census Block. The population was calculated for each Census Track and then aggregated into each county. 

This study found that both decreasing chute time and increasing the travel speed over the roads can significantly improve the EMS through urban, rural, and frontier communities. This study recommends that public health planners, transportation engineers, and policy makers focus on improvement of chute time in urban settings and improvement of infrastructure and operational service for emergency vehicles to speed up while maintaining safety on roads. The study demonstrated that visualizing the backup service coverage and the population covered ratio show ease of use and usefulness to provide impactful information to the public and the public health planners. 

However, several points should be carefully addressed to translate the results into application. As the parameters for the chute time and operating vehicle speed of the scenarios adopted in this study can vary by different conditions, additional sensitivity analysis is required to align with the local and state situations and level of demand in actual public health plans. In addition, the regional categories should be acceptable by the public. Emerging technologies and sources such as GPS and real-time tracking devices can be utilized to estimate the vehicle speed on roads. The 911 calls of locations are assumed based on the centroid of Census Block, but the data can be collected for better planning. This study focused on the response time from the ambulance base to a scene, but the transport time from the scene to a hospital should be investigated to save patient’s life with the destined hospital’s capacity. Results of this study should build on the transport time from the scene to the hospital. 

## Figures and Tables

**Table 1:** Chute time in minutes in Midwest refer to NEMSIS (95% Confidence Interval).

Region in the Midwest	Organization Status	Median	Lower Limit	Upper Limit	N
Urban/Suburban	Volunteer	4.87	4.82	4.91	3993
	Non-Volunteer	2.50	2.45	2.52	5250
Rural	Volunteer	5.50	5.41	5.64	1754
	Non-Volunteer	3.86	3.80	3.94	1993
Wilderness (frontier)	Volunteer	6.25	6.13	6.33	2113
	Non-Volunteer	4.00	3.39	4.00	2200
Overall (95% Bootstrap CI: Percentile Method)	Volunteer	4.89	4.84	4.93	2844
	Non-Volunteer	4.12	4.06	4.16	2844

**Table 2:** Assumed travel speed ambulance.

MTFCC	Description		Scenario 1 & 3	Scenario 2 & 4
			Speed Estimated (Miles per Hour)	Speed Assumed for Ambulance
S1100	Primary roads with limited access on highways	Rural	75	80
		Urban	55	60
S1200	Secondary roads (US highway, State highway, county highways)	Paved and divided multilane	70	75
		Paved two-lane	65	70
S1400	Local neighborhood road, rural road, city street; paved non-arterial		55	60
S1500	Vehicle trail (4WD)		25	25
S1630	Ramp		25	25
S1640	Service drive usually along a limited access highway		25	30
S1740	Private road for service vehicles (logging, oil fields, ranches, etc.)		25	30

Note: MTFCC stands for the MAF/TIGER Feature Class Code.

**Table 3:** Chute time assumption and travel time for each geographical category.

Scenarios	Location of An Ambulance	Chute Time (Minutes)	Recommended Drive Time (Minutes)		
			$\mathrm{Urban}\;\left(RT_{o}^{R=1}=9\right)$	$\mathrm{Rural}\;\left(RT_{o}^{R=2}=20\right)$	$\mathrm{Frontier}\;\left(RT_{o}^{R=3}=30\right)$
Scenario 1 and 2	Urban $\left(j\in R1\right)$	CT_R=1_ = 2.5	d*_11_= [0.00–6.50]	d*_12_ = [0.00–17.50]	d*_13_ = [0.00–27.50]
	Rural $\left(j\in R2\right)$	CT_R=2_ = 5.5	d*_21_ = [0.00–3.50)	d*_22_ = [0.00–14.50]	d*_23_ = [0.00–24.50]
	Frontier $\left(j\in R3\right)$	CT_R=3_ = 6.25	d*_33_ = [0.00–2.57]	d*_32_ = [0.00–13.75]	d*_33_ = [0.00–23.75]
Scenario 3 and 4	Urban $\left(j\in R1\right)$	CT_R=1_ = 0.5	d*_11_ = [0–8.5]	d*_12_ = [0.0–19.5]	d*_13_ = [0–29.5]
	Rural $\left(j\in R2\right)$	CT_R=2_ = 4.95	d*_21_ = [0–4.05]	d*_22_ = [0–15.05]	d*_23_ = [0–25.05]
	Frontier $\left(j\in R3\right)$	CT_R=3_ = 5.62	d*_31_ = [0–3.38]	d*_32_ = [0–14.38]	d*_33_ = [0–24.38]

Note: $d_{j\in R,i\in R}^{*}$ denotes recommended travel time to meet the required response time.

**Table 4:** Population and land coverage by each scenario for each geographic region.

Region	Scenarios	Changes	Population (Person)			Land (Square Miles)		
			Sum	Covered	Ratio	Sum	Covered	Ratio
Frontier *P*_*R*=2_(%)	Scenario 1	As-Is	105,889	103,368	97.6%	35,616	31,306	87.9%
	Scenario 2	Speed ↑	105,889	104,686	98.9%	35,616	33,237	93.3%
	Scenario 3	Chute ↓	105,889	103,721	98.0%	35,616	31,806	89.3%
	Scenario 4	Speed ↑ & Chute ↓	105,889	104,876	99.0%	35,616	33,576	94.3%
Rural *P*_*R*=1_(%)	Scenario 1	As-Is	182,667	159,054	87.1%	34,614	20,772	60.0%
	Scenario 2	Speed ↑	182,667	165,194	90.4%	34,614	23,621	68.2%
	Scenario 3	Chute ↓	182,667	163,034	89.3%	34,614	22,384	64.7%
	Scenario 4	Speed ↑ & Chute ↓	182,667	169,300	92.7%	34,614	25,238	72.9%
Urban *P*_*R*=0_(%)	Scenario 1	As-Is	384,035	350,271	91.2%	483	327	67.7%
	Scenario 2	Speed ↑	384,035	368,065	95.8%	483	376	77.8%
	Scenario 3	Chute ↓	384,035	377,722	98.4%	483	408	84.5%
	Scenario 4	Speed ↑ & Chute ↓	384,035	382,504	99.6%	483	429	88.8%
Total *P*(%)	Scenario 1	As-Is	672,591	612,693	91.1%	70,713	52,405	74.1%
	Scenario 2	Speed ↑	672, 591	637,945	94.8%	70,713	57,235	80.9%
	Scenario 3	Chute ↓	672, 591	644,477	95.8%	70,713	54,598	77.2%
	Scenario 4	Speed ↑ & Chute ↓	672, 591	656,680	97.6%	70,713	59,243	83.8%

## Data Availability

The data that support the findings of this study are available from the public sources: ambulance service location in North Dakota is available from the ND GIS Hub (https://gishubdata.nd.gov/dataset/ambulance-service-locations) (accessed on 14 February 2021). Census Blocks of 2010 is available from the U.S. Census Bureau (https://www2.census.gov/geo/tiger/TIGER2010/TABBLOCK/2010/) (accessed on 14 February 2021). The raw data of frontier and remote area codes are available from the U.S. Department of Agriculture (https://www.ers.usda.gov/data-products/frontier-and-remote-area-codes/frontier-and-remote-area-codes/#2010%20Frontier%20and%20Remote%20Area%20Codes%20Data%20Files) (accessed on 14 February 2021). The state and local road networks of North Dakota are available from the U.S. Department of Commerce and North Dakota GIS Hub (https://gishubdata.nd.gov/dataset/tiger-roads) (accessed on 14 February 2021).
